# Does Pre-Existing Chronic Obstructive Pulmonary Disease Increase the Risk of Checkpoint Inhibitor Pneumonitis in Advanced/Metastatic Non-Small Cell Lung Cancer Treated with Immune Checkpoint Inhibitors?

**DOI:** 10.3390/curroncol32050259

**Published:** 2025-04-29

**Authors:** David Spillane, Carmela Pepe, Goulnar Kasymjanova, Diane Cruiziat, Sara Cohen, Jeremy Naimer, Jason Agulnik

**Affiliations:** 1 Anna and Peter Brojde Lung Cancer Centre, Jewish General Hospital, Montreal, QC H3T 1E2, Canada; david.spillane.med@ssss.gouv.qc.ca (D.S.); carmela.pepe.med@ssss.gouv.qc.ca (C.P.); jason.agulnik.med@ssss.gouv.qc.ca (J.A.); 2 Department of Medicine, McGill University, Montreal, QC H3A 0C7, Canada; 3 Anatomy & Cell Biology, Downtown Campus, McGill University, Montreal, QC H3A 0C7, Canada; diane.cruiziat@mail.mcgill.ca (D.C.); sara.cohen2@mail.mcgill.ca (S.C.); jeremy.naimer@mail.mcgill.ca (J.N.)

**Keywords:** NSCLC, immune checkpoint inhibitors, checkpoint inhibitor pneumonitis

## Abstract

Objective: Immune checkpoint inhibitors (ICIs) are front-line treatment options for NSCLC. ICI therapy is associated with a risk of immune-related adverse events (irAEs). Checkpoint inhibitor pneumonitis (CIP) is a potentially life-threatening irAE. Previous studies have demonstrated that asthma and interstitial lung disease are associated with an increased risk of CIP. We sought to determine whether chronic obstructive pulmonary disease (COPD) is associated with CIP. Methods: This retrospective study examines a cohort of ICI-treated NSCLC patients either with or without chemotherapy at the Anna and Peter Brojde Lung Cancer Centre, Jewish General Hospital in Montreal, Canada between 2014 and 2023. We explored associations between risk factors and CIP using the Mann–Whitney U test or Fisher’s exact test. Analysis of prognostic factors was performed using a logistic regression model. All statistical analyses were carried out using SPSS software, version 24.0 (SPSS, Chicago, IL, USA). *p*-values of 0.05 or less were considered significant. Results: Of the 327 selected patients on ICIs, 23 experienced an acute respiratory deterioration that was attributed to CIP, 87/327(26.6%) patients had a pre-existing diagnosis of COPD, and 11/87 (12.6%) COPD patients experienced CIP compared to 13/240 (5.5%) non-COPD patients (*p* = 0.061). There was no statistical or clinically meaningful correlation between COPD severity and CIP. The only variable significantly associated with CIP was a poor ECOG performance status. Among ECOG 1 patients, 18/91 (19.8%) experienced CIP compared to 5/226 (2.2%) of those with an ECOG of 0. A multivariate assessment involving all 327 patients revealed no significant factors affecting CIP development. Conclusions: Our single-institution study revealed that although there was a trend, the presence of COPD was not statistically associated with an increased risk of CIP. Additionally, neither FEV1 nor DLCO had a meaningful impact on the development of CIP in COPD patients. Given these findings, we emphasize the need for larger prospective studies to confirm these observations before drawing definitive clinical recommendations.

## 1. Introduction

COPD is a highly prevalent comorbidity among patients with NSCLC, largely due to shared risk factors, such as smoking and aging. The condition affects an estimated 30–70% of this population, depending on the diagnostic criteria and study demographics. It is characterized by long-term respiratory symptoms and airflow limitations, which not only impact overall patient health but also pose unique challenges in lung cancer treatment. [1,2,3] Diagnosis typically relies on spirometry, with additional tools such as chest X-rays or CT scans for assessing lung damage, arterial blood gas analysis, and genetic testing for alpha-1 antitrypsin deficiency [1,2].

Given the high prevalence of COPD among lung cancer patients and its impact on overall health, treatment strategies must address the challenges posed by this condition. Immune checkpoint inhibitors (ICIs) have revolutionized the management of NSCLC, offering significant survival benefits, particularly for advanced-stage disease. Monotherapy ICIs represent groundbreaking front-line treatment options for EGFR/ALK/ROS1 non-mutant non-small cell lung cancer (NSCLC) with high PDL1 expression (≥50% expression) [4,5,6,7]. For patients exhibiting lower PDL1 expression levels, ICIs combined with traditional chemotherapy demonstrate a marked enhancement in clinical outcomes. Studies suggest that the incidence of CIP varies among different ICIs. Nishino et al. have performed a meta-analysis of 26 studies and reported on the incidence of CIP. They stated that PD-1 and PD-L1 inhibitors were more likely to induce CIP rather than CTLA-4 inhibitors [8]. Other authors have reported that the incidence of CIP was higher with combination immunotherapy vs. monotherapy.

However, the interplay between pre-existing respiratory conditions, such as COPD, and the potential pulmonary toxicity associated with ICIs, such as CIP, presents unique challenges that warrant careful consideration [9]. CIP can manifest with a wide spectrum of symptoms, ranging from mild to severe, with common symptoms being cough, shortness of breath (dyspnea), chest pain, and fever. Diagnosing CIP requires imaging, such as chest CT scans, and careful exclusion of other causes, like infections, heart failure, or tumor progression. Reported incidence rates vary by treatment, with PD-1 and PD-L1 inhibitors associated with a frequency below 10%, while CTLA-4 inhibitors show even lower rates [10,11,12,13,14,15]. Differentiating CIP from other conditions, like infections or cardiac issues, can require invasive diagnostics (e.g., bronchoscopy or biopsy), which may not always be feasible in COPD patients with reduced lung function. Management strategies typically involve the discontinuation of ICIs and corticosteroid therapy, with permanent discontinuation necessary in severe cases [9]. Grades of CIP based on the Common Terminology Criteria for Adverse Events (CTCAE) may range from asymptomatic (Grade 1) to life threatening or death (Grades 4 and 5). Understanding whether COPD exacerbates the risk of CIP is critical to optimizing treatment strategies for this vulnerable population.

The research questions are as follows: Does pre-existing COPD increase the risk of CIP in metastatic NSCLC treated with ICIs (either as alone or in combination with systemic chemotherapy)? Should a diagnosis of COPD be considered during treatment decision making?

## 2. Materials and Methods

This retrospective study examines a cohort of NSCLC patients who underwent ICI treatment, either with or without chemotherapy, at the Anna and Peter Brojde Lung Cancer Centre, Jewish General Hospital in Montreal, Canada between 2014 and 2023. Eligible patients, identified from the outpatient oncology clinic, were stage 4, had an ECOG-PS of 0–1, and a life expectancy of at least 4 months at the start of therapy. This study was approved by the Research Ethics Board (REB).

Pre-existing diagnoses of COPD were identified through a thorough review of patients’ medical histories. This process involved analyzing clinical documentation from pulmonologists and oncology specialists, as well as reviewing past hospital records and discharge summaries for evidence of a COPD diagnosis.

For a more objective assessment, available pulmonary function test (PFT) data were included to confirm and characterize the severity of COPD. Forced expiratory volume in one second (FEV1) and diffusing capacity for carbon monoxide (DLCO) were analyzed when available, providing further insight into the extent of pulmonary impairment. In this study, we investigated the statistical significance of COPD parameters in the CIP and non-CIP groups. In addition, to measure the real-world impact of the FEV1 and DLCO differences on the development of CIP, we investigated minimal clinically important differences (MCIDs) by calculating Cohen’s d statistic. Cohen’s d < 0.5 is considered small and may not be meaningful [16]. This approach ensured a comprehensive understanding of the pulmonary health of the cohort, which was crucial for investigating the relationship between COPD and the incidence of CIP.

CIP diagnosis involved a meticulous retrospective review of charts, focusing on the term ‘pneumonitis’. In our study, we focused on Grade 2+ CIP, which excludes other causes of respiratory distress such as infection, disease progression, and heart failure through laboratory tests, imaging, and bronchoscopy if needed. Starting in 2014, the involvement of a multidisciplinary tumor board (including oncologists, pulmonologists, and radiologists) was absolutely necessary to reach a consensus diagnosis of CIP.

The baseline characteristics of patients who experienced CIP were compared with those who did not. Statistical tests, including Mann–Whitney U and logistic regression, were selected to ensure robust analysis of continuous and categorical variables. Logistic regression analysis was employed to estimate odds ratios (ORs) in order to investigate the risk factors associated with CIP. The model included covariates such as COPD, PD-L1 status, the choice between ICI monotherapy and combination with chemotherapy (CTX), Eastern Cooperative Oncology Group Performance Status (ECOG PS), smoking history, and histology. All *p*-values were two sided, and those equal to or less than 0.05 were considered statistically significant. Statistical analyses were conducted using SPSS software, version 24.0 (SPSS, Chicago, IL, USA).

## 3. Results

During the study period, 327 patients diagnosed with metastatic NSCLC underwent treatment with ICIs, either alone or in conjunction with CTX. Patient characteristics are presented in Table 1. Their mean age was 69 years, with men constituting 48% of the cohort. A majority of patients were former or current smokers (81.3%) and had an ECOG PS of 0 (73.6%). Non-squamous and squamous NSCLC was diagnosed in 73% and 27% of patients, respectively. The majority of patients (71%) received pembrolizumab-based therapy, either as monotherapy or in combination with chemotherapy (CTX). Nivolumab was administered to 24% of patients. Concerning PD-L1 expression, 291 (89%) patients had their PD-L1 status determined, while in 36 cases (11%), it could not be quantified due to either insufficient tissue or inadequate quality. Of those with known PD-L1 status, 152 (52.2%) patients had a Tumor Proportion Score (TPS) of 50% or higher, 93 (31.9%) exhibited a TPS of 1–49%, and 46 (15.9%) had a TPS of less than 1%. Among the 327 study patients, 23 (7.0%) had an MTB consensus diagnosis of Grade 2+ CIP, and 304/327 (93%) were considered non-CIP. A total of 19/304 (6.3%) patients in the non-CIP group experienced a respiratory symptom that could not clearly be attributed to Grade 2+ CIP and did not require the discontinuation of ICIs and corticosteroid therapy. We conducted a comparative analysis of baseline characteristics between patients who developed Grade 2+ CIP and those who did not, as shown in Table 1. Despite the limited sample size, our investigation demonstrated a significant difference in the incidence of CIP in patients with an ECOG PS of 1, with a rate of 19.8%, whereas those with an ECOG PS of 0 demonstrated a lower occurrence of 2.1% (*p* = 0.001). We noted a trend, although not statistically significant, of an increased incidence of CIP among former and current smokers, high PD-L1 expressers, squamous histology, patients with COPD, and those receiving pembrolizumab-based therapy.

Pre-existing COPD was found in 87/327 (28.7%) patients. Among those, 10/87 (11.5%) developed CIP compared to 13/240 (5.4%) of non-COPD patients (*p* = 0.061).

PFTs were available for 74/86 (86%) of patients with pre-existing COPD. Out of those, 16/74 (21.6%) exhibited severe and very severe COPD, 48 (65%) exhibited moderate COPD, and 10 (13.4%) showed mild disease (Table 2).

PFT data were available for 9/10 (90%) of COPD patients who developed CIP. Among these, one patient had severe, six had moderate, and two had mild COPD. There was no significant correlation between the severity of COPD and the incidence of CIP, although the numbers were small.

Table 3 reveals differences in FEV1 and DLCO values between patients who developed CIP and those who did not.

- FEV1: The mean FEV1 was slightly lower in CIP patients (1.9061 ± 0.7643) compared to non-CIP patients (1.9875 ± 0.6406). However, the effect size, measured using Cohen’s d, was 0.12, indicating a negligible difference.
- DLCO: The mean DLCO was higher in CIP patients (15.857 ± 5.7724) than in non-CIP patients (13.253 ± 6.5029). The effect size for DLCO was 0.42, suggesting a small to moderate difference.

These findings highlight that while there may be a trend toward differing baseline lung function between CIP and non-CIP groups, the clinical significance of these differences requires further exploration in larger cohorts.

**Table 3:** Minimal clinically important difference (MCID) of the COPD effect.

		Mean	SD	Effect Size (Cohen’s *d*)
FEV1	CIP	1.9061	0.7643	0.12
	Non-CIP	1.9875	0.6406	
DLCO	CIP	15.857	5.7724	0.42
	Non-CIP	13.253	6.5029	

Analyses of prognostic factors were performed using a logistic regression model (Table 4). The variables included in the model are listed below. Regression analysis, which included all 327 patients, showed that none of the analyzed factors were statistically significant in CIP development. The presence of COPD showed a trend toward increased risk; however, the confidence interval was wide (0.92–4.68), and the *p*-value did not reach statistical significance (0.079).

## 4. Discussion

In this retrospective chart review study, we analyzed risk factors for CIP in 327 advanced NSCLC patients, with a particular focus on COPD as a possible risk factor. According to Health-InfoBase Canada, around 10% of Canadians aged 35 years and older live with COPD. The incidence rate of COPD among former smokers is roughly one in four, and among current smokers, it is approximately one in two individuals [17]. It is important to note that the likelihood of having COPD increases with age [18]. In our study, we found that 27% of our total population had a history of pre-existing COPD. This figure is higher than expected, which could be attributed to the fact that 81.3% of our patients were current or former smokers, and the mean age of the cohort is 69.

The overall incidence of CIP in our cohort was 7.0%, which is similar to the previously published results of clinical trials, as well as some real-world studies [13,19,20,21]. In the univariate analysis, the rate of CIP was higher in patients with pre-existing COPD (11.5%) compared to those with no history of COPD (5.5%); however, this difference was not statistically significant. We recognize that the small sample size might limit the ability to detect statistically significant associations. However, the observed statistical difference of 0.061 might suggest a potential impact of COPD in the development of CIP, highlighting the need for further larger, multicenter prospective studies. A statistically significant association was observed between ECOG PS and CIP incidence. Patients with ECOG PS 1 had a notably higher rate of CIP (18/23) compared to those with ECOG PS 0 (5/23). This finding aligns with Aiad et al., who reported that an increased ECOG PS score was independently associated with an increased risk factor for CIP [22]. These results suggest that functional status may influence CIP risk, possibly due to differences in baseline lung function, systemic inflammation, or the ability to tolerate immune-related toxicities.

Multivariate logistic regression analysis did not identify any statistically significant predictors of CIP. COPD was associated with an increased risk (OR = 2.27, 95% CI: 0.924–4.68, *p* = 0.079), but the confidence interval was wide, and statistical significance was not reached. These highlight the need for larger sample sizes to clarify potential risk factors for CIP more accurately.

Additionally, we evaluated pulmonary function test parameters of CIP, especially FEV1 and DLCO, in COPD patients with or without CIP. The difference in mean FEV1 and DLCO between the two groups had a small to moderate effect size (0.12 and 0.42, respectively). However, MCID has not been well studied in this population, and the clinical implication of these findings remains unclear [16]. Further research is needed to determine whether COPD severity influences CIP risk.

Studies exploring the relationship between COPD and CIP have produced conflicting results. Our findings align with Mark et al., who conducted a prospective study on COPD and CIP risk in NSCLC patients receiving ICIs. They found that while COPD patients exhibit stronger pro-inflammatory characteristics, they still benefit from ICI therapy without a significant increase in CIP incidence [23]. Similarly, M. Altan et al. reported a CIP of 9.5% (40/419) among metastatic NSCLCs but found no association between COPD and CIP [24]. Zhu Zeng et al. also found that COPD did not predict CIP risk in patients with NSCLC patients receiving ICIs [25]. These studies support the continued use of ICIs in COPD patients, reinforcing that COPD should not be contraindicated with immunotherapy.

In contrast, F. Li et al. conducted a meta-analysis of six studies evaluating CIP risk in NSCLC patients receiving ICIs. It revealed significant heterogeneity, with three studies reporting an increased CIP risk in COPD patients and three finding no association. Pooled analysis suggested that COPD patients had a higher probability of developing CIP [26]. The authors hypothesized that chronic inflammation in COPD may contribute to CIP, potentially by increasing pulmonary immune responses. This inconsistency in findings underscores the need for further research to clarify the COPD-CIP relationship.

Our study has several limitations. First, it is important to note that our study was retrospective in nature, relying on historical data and medical records. Second, PFT data were available only for 86% of COPD patients, limiting our ability to fully characterize COPD severity. Third, CIP diagnosis remains challenging since it often relies on excluding other possible conditions, and misidentification may occur in cases with overlapping infection or inflammatory conditions. Future prospective studies with larger sample sizes are needed to validate these findings. Real-time data collection, standardized diagnostic criteria, and a more comprehensive assessment of COPD severity could help better define the impact of COPD on CIP risk.

## 5. Conclusions

Our single-institution study revealed that although there was a trend, the presence of COPD was not statistically associated with an increased risk of CIP. Additionally, neither FEV1 nor DLCO had a meaningful impact on the development of CIP in COPD patients. Given these findings, we emphasize the need for larger prospective studies to confirm these observations before drawing definitive clinical recommendations.

## Figures and Tables

**Table 1:** Study cohort characteristics.

Characteristics		Total N = 327	Non-CIP ^1^ N = 304	CIP N = 23	*p*-Value
Age (mean range)		69 (27–95)	69 (27–91)	70 (52–85)	0.798
Sex (n/%)	Male	158 (48.0)	147 (93.0)	11 (7.0)	0.984
	Female	169 (52.0)	157 (92.0)	12 (8.0)	
Smoking history (n/%)	Former/current smoker	266 (81.3)	245 (92.0)	21 (8.0)	0.272
	Non-smoker	61 (19.7)	59 (96.0)	2 (4.0)	
ECOG PS (n/%)	0	236 (73.6)	231 (97.9)	5 (2.1)	0.001
	1	91 (26.4)	73 (80.2)	18 (19.8)	
COPD (n/%)	Present	87 (28.7)	77 (88.5)	10 (11.5)	0.061
	Absent	240 (71.3)	227 (94.5)	13 (5.5)	
PD-L1 (n/%)	≥50	152 (52.2)	139 (91.2)	13 (8.8)	0.741
	1–49	93 (31.9)	85 (90.6)	8 (9.4)	
	<1	46 (15.9)	44 (94.7)	2 (5.3)	
Histology	Non-squamous	234 (73)	220 (94.0)	14 (6.0)	0.172
	Squamous	93 (27)	84 (90.3)	9 (9.7)	
Immunotherapy type	Pembrolizumab+/−CTX ^2^	231 (71)	218 (94.3)	13 (5.7)	0.067
	Nivolumab	80 (24)	73 (78.7)	7 (21.3)	
	Other	15 (5)	12 (80)	3 (20)	

^1^ Checkpoint inhibitor pneumonitis; ^2^ chemotherapy.

**Table 2:** Severity of COPD at the baseline.

PFT Parameters	% Predicted	Non-CIP	CIP	*p*-Value
FEV1 (n = 74) ^1^	>80% (mild)	8 (12.03)	2 (22.2)	0.695
	50–79% (moderate)	42 (64.6)	6 (66.7)	
	30–49% (severe)	11 (16.9)	1 (11.1)	
	<30% (very severe)	4 (6.2)	0	
DLCO ^2^ (n = 57) ^3^	≥75%	13 (34.0)	4 (66.7)	0.135
	<75	31 (61.0)	2 (33.3)	

^1^ Twelve patients were missing FEV1 (forced expiratory volume in 1 s) data; ^2^ diffusing capacity of the lungs for carbon monoxide; ^3^ twenty-nine patients were missing DLCO data.

**Table 4:** Logistic regression analysis of predictive factors for CIP.

Variables	OR ^1^	95% CI	*p*-Value
Age	1.02	0.832–1.540	0.910
Sex	0.982	0.519–1.265	0.614
ECOG PS	0.095	0.260–1.260	0.478
Smoking status	0.435	0.363–1.710	0.470
Histology	1.670	0.676–3.108	0.132
COPD	2.270	0.924–4.680	0.079
IO type	0.548	0.203–1.567	0.370

^1^ Odds ratio.

## Data Availability

Data are not publicly available due to ethical restrictions.
